# An Exploratory Study of Training Intensity in EEG Neurofeedback

**DOI:** 10.1155/2021/8881059

**Published:** 2021-03-11

**Authors:** Inês Esteves, Wenya Nan, Cristiana Alves, Alexandre Calapez, Fernando Melício, Agostinho Rosa

**Affiliations:** ^1^Evolutionary Systems and Biomedical Engineering Lab, Institute for Systems and Robotics, Instituto Superior Técnico, University of Lisbon, 1049-001 Lisbon, Portugal; ^2^Department of Psychology, Shanghai Normal University, Shanghai 200234, China; ^3^Instituto Superior de Engenharia de Lisboa (ISEL), Instituto Politécnico de Lisboa, 1959-007 Lisbon, Portugal; ^4^Department of Bioengineering, Instituto Superior Técnico, University of Lisbon, 1049-001 Lisbon, Portugal

## Abstract

Neurofeedback training has shown benefits in clinical treatment and behavioral performance enhancement. Despite the wide range of applications, no consensus has been reached about the optimal training schedule. In this work, an EEG neurofeedback practical experiment was conducted aimed at investigating the effects of training intensity on the enhancement of the amplitude in the individual upper alpha band. We designed INTENSIVE and SPARSE training modalities, which differed regarding three essential aspects of training intensity: the number of sessions, the duration of a session, and the interval between sessions. Nine participants in the INTENSIVE group completed 4 sessions with 37.5 minutes each during consecutive days, while nine participants in the SPARSE group performed 6 sessions of 25 minutes spread over approximately 3 weeks. As a result, regarding the short-term effects, the upper alpha band amplitude change within sessions did not significantly differ between the two groups. Nonetheless, only the INTENSIVE group showed a significant increase in the upper alpha band amplitude. However, for the sustained effects across sessions, none of the groups showed significant changes in the upper alpha band amplitude across the whole course of training. The findings suggest that the progression within session is favored by the intensive design. Therefore, based on these findings, it is proposed that training intensity influences EEG self-regulation within sessions. Further investigations are needed to isolate different aspects of training intensity and effectively confirm if one modality globally outperforms the other.

## 1. Introduction

Neurofeedback (NF) relies on the voluntary modulation of brain activity where brain signals are extracted and evaluated in real time and presented back to the individual in the form of an auditory or visual feedback [1]. NF is non-invasive and safe and has potential to modulate brain activity for cognitive and behavioral enhancement [2]. Therefore, it reveals a great potential, as a complementary or alternative therapy, to deal with physical or mental disorders (e.g., attention-deficit/hyperactivity disorder (ADHD) [3, 4], autism spectrum disorder (ASD) [5], and schizophrenia [6]), when the conventional treatments are not successful or induce negative side effects. In addition to clinical applications, in recent years, research has spread to nonmedical fields and newer protocols have been applied [7, 8].

However, despite the increasing widespread use of this technique, some aspects of NF methodology, such as the number of frequency bands, feedback modality, number of electrodes, and training intensity, are not standardized [9]. Training intensity comprehends aspects such as the number of sessions, the duration of a session, and the spread of the training over time. For the same application, very different training intensity parameters have been used. For instance, regarding clinical purposes, Marzbani et al. [2] report a number of sessions ranging from 21 to 100 for autism spectrum disorder and from 18 to 40 for ADHD, while for the improvement of sports performance, Mirifar et al. [8] present studies with a number of sessions ranging from 1 to 20.

NF learning refers to gaining control over brain activity, by adjusting either a band amplitude, frequency, connectivity, or other features that characterize it. Regardless of the success of NF in many cases, the effectiveness of NF treatment is often variable among subjects. A significant proportion of subjects reveals to be unable to achieve control over brain metrics, even following several training sessions. These subjects are usually considered “non-learners,” in opposition to “learners.” Due to the key role of NF learning on the improvement of a target behaviour or cognition, the failure to achieve the desired control over brain activity means that the subjects may not benefit from the NF treatment, which represents a critical issue [10]. Several authors attribute poor or unexpected results to insufficient training time [11–13]. Also, when assessing NF learning, it might even be the case that non-learners, i.e., subjects that do not respond to the protocol as expected, simply need more sessions to consolidate results [14]. As there is no optimal protocol regarding intensity, it may also be useful to understand, for example, if the learning process may occur within a short period as effectively as during a long one. Short-period training is expected to be more easily accepted by some participants as it is lighter and takes less effort regarding scheduling. Therefore, deeper knowledge of training intensity effects could help to design a more effective training.

Rogala et al. [9] defined a training intensity index based on the total number of training days and the intervals between sessions. By reviewing studies with healthy participants, they concluded that the EEG NF training sessions were usually composed of several periods of a few minutes with short pauses between them. Furthermore, for their sample of 28 experiments, the NF training consisted, on average, of 7.7 ± 3.8 sessions, separated by 3 ± 2.4 days, being less intensive than the training implemented in clinical studies, which is usually up to 30 or 40 sessions [15]. However, they did not find a significant dependency between training success and training intensity. From the 28 examined experiments, 23 used single EEG band protocols, and most of those were intended to upregulate the amplitude of the target band, which included theta, alpha, beta, gamma, and slow cortical potentials (SCP). The other 5 experiments employed a multiband protocol aimed at changing the ratio between the amplitudes of two bands.

In this work, an experiment of EEG NF was performed with healthy participants to test if contrasting training intensity with the same total NF time (distinct with respect to the number of sessions, the interval between sessions, and the duration of each session) produces significant differences. Additionally, to motivate the participants, the chosen protocol was upper alpha (UA) band NF, aiming to improve their working memory [16]. Our focus is on studying the learning of EEG regulation (i.e., NF learning), which in this case is learning to increase the amplitude of the UA band, and how it is affected by training intensity. The NF learning was assessed by examining the changes in amplitude of UA across sessions and within session.

## 2. Methods

### 2.1. Participants

A total of 19 healthy subjects participated in this study. Participants were allocated to two different groups: INTENSIVE (*n* = 9) and SPARSE (*n* = 10). The participants were not randomly assigned to their group due to the significant difference in the training load of the two training intensity modalities. Time constraints had to be taken into account, and thus, the choice was made according to participants' requirements considering their availability. There was one dropout in the SPARSE group after 4 sessions, due to incompatibilities with personal schedule. The following exclusion criteria were used: age (minors were not allowed), severe health problems or psychological disorders, abnormal cortical activity detected in the EEG, significant skull/brain damage, and intake of psychotropic drugs that could significantly alter brain function and consciousness. None of the participants reported major health issues. Two of the participants (one in the INTENSIVE group and one in the SPARSE group) had previously performed NF training, yet with a distinct protocol. Considering the final sample, the INTENSIVE group consisted of 2 males and 7 females (age: 23.44 ± 2.41 range: 22-30) while the SPARSE group consisted of 6 males and 3 females (age: 27.67 ± 9.81, range: 22-46). The Wilcoxon rank-sum test showed no significant difference between groups regarding age (*U* = 38.5, *p* = 0.857). The chi-square test showed no significant difference in gender (*χ*^2^(1) = 3.6, *p* = 0.058) between the two groups.

All participants gave written informed consent before the experiment started. The protocol was in accordance with the Declaration of Helsinki and approved by the Ethics Committee of the Centro Hospitalar Lisboa Norte and Centro Académico de Medicina de Lisboa. Participants were all volunteers, and no monetary reward was given for their cooperation with this study.

### 2.2. Design

**Figure 1:**
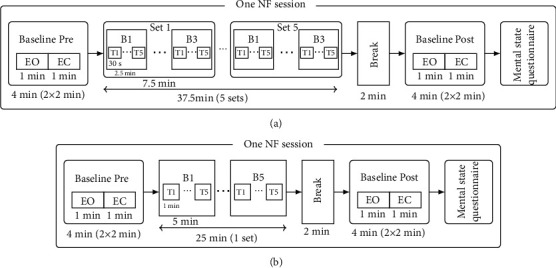
(a) Session diagram for the INTENSIVE group. (b) Session diagram for the SPARSE group. Each session is organized in blocks (B), which are composed of trials (T); for the baseline and pre- and post-NF, both eyes open (EO) and eyes closed (EC) were recorded.

The difference between INTENSIVE and SPARSE groups was only training intensity. The INTENSIVE group performed 4 sessions, with 37.5 min of NF each, in 4 consecutive days, while the SPARSE group performed 6 sessions, with 25 min of NF each, spread along approximately 3 weeks, with 2 to 4 sessions per week. Therefore, each group had 150 minutes of NF training totally. The NF periods in each session were organized in sets of blocks, with each block consisting of several trials. The plan for each session is depicted on Figures 1(a) and 1(b) (for the INTENSIVE and SPARSE groups, respectively).

Besides NF training (upregulation of the UA amplitude with feedback), both groups were also submitted to transfer trials (upregulation of the UA without feedback) and cognitive tests (tests to assess working memory performance). However, these results will not be analyzed in this paper, since the focus is on NF learning, and practice effects of the behavioural tests cannot be ruled out without a control group. Resting baselines were preceded by a 2-minute relaxed state and assessed in both eyes open (EO) and eyes closed (EC) conditions. Two alternating epochs of 1 minute each were recorded for each condition, both at the beginning and at the end of each session. The 36-Item Short Form Survey (SF-36) questionnaire was used to assess the general health state in the first session (before training). In every session, participants were also asked to fill in a questionnaire in which they rated several parameters referring to their mental state during the session.

### 2.3. Signal Acquisition

The acquisitions were carried out using Somnium software [17], in a room provided by the Evolutionary Systems and Biomedical Engineering Lab (LaSEEB), a research lab of the Institute for Systems and Robotics (ISR), at Instituto Superior Técnico (IST), University of Lisbon. Electrodes were placed according to the International 10-20 System, using the left and right mastoids as references for common mode rejection and the middle of the forehead as ground. Relevant signal was recorded, with a sampling frequency of 250 Hz, from 20 electrodes: Fz, Fp1, F7, F3, T3, C3, T5, P3, O1, Cz, Pz, Oz, Fp2, F8, F4, T4, C4, T6, P4, and O2, and amplified by the EEG amplifier Vertex 823 (produced by Meditron Electromedicina Ltda, São Paulo, Brazil), with an analog bandpass filter between 0.1 and 70 Hz. The impedance of each electrode was kept below 10 k*Ω*. Participants were asked to remain as still as possible and also to avoid excessive blinking and abrupt movements.

### 2.4. Protocol

Although the main focus of the present work was to study the training intensity effects on NF learning, an NF protocol for enhancement of working memory was used so that healthy participants felt more engaged with the tasks. For that purpose, the training was aimed at enhancing UA amplitude at Fz, as both the UA and the frontal area are associated with memory functions. On the one hand, the choice of a frontal region was based on the important role that the prefrontal cortex plays in working memory [18, 19]. We opted for an electrode placed in the frontal midline in order to avoid changing frontal asymmetry which could impact affective processing [20]. On the other hand, the evidence of a link between increased UA activity and good working memory performance [16, 21–23] led to the decision of training individuals to increase the activity within this band. Furthermore, previous NF studies had already used it with the goal of improving cognitive performance, having successfully enhanced working memory [1, 16, 24, 25]. Finally, given that alpha frequency has large interindividual difference [26], this study trained the amplitude in the individual UA band instead of the fixed UA band.

#### 2.4.1. Individual Upper Alpha Measurement

The first baseline measurements of the first session were used to define the individual alpha band (IAB) of each individual, based on the difference between EO and EC spectra [27, 28]. The signals were submitted to notch filtering (50 Hz) and low pass filtering (30 Hz), and the power spectrum density was estimated using Welch's method [29], with an overlap of 10% and a segment length of 5 seconds. The crossings between EO and EC spectra provided the frequency boundaries: Lower Transition Frequency (LTF) and Higher Transition Frequency (HTF) [13]. If the crossings were not clearly visible from the spectra at Fz, we investigated occipital electrodes, where the alpha activity is usually more pronounced [30]. The individual UA band was defined as the frequency range between the individual peak alpha frequency and the HTF. The individual peak alpha frequency was defined as the frequency with the largest power in the range 7.5-12.5 Hz in the EC power spectra and was considered equivalent to the Individual Alpha Frequency (IAF). The frequency range obtained for each participant was used for the online feedback and the subsequent offline analysis.

#### 2.4.2. Neurofeedback Training

*(1) Feedback*. The EEG training platform integrated in the Somnium software was adopted to perform NF training, using a visual feedback modality with the display described in more detail in [17]. This display uses two three-dimensional objects against a grey background: a white/purple sphere, in the center, and a blue cube, in the lower left corner. These shapes suffer changes during the training, reacting to the participant's EEG in real time, according to previously defined settings. If the feedback parameter surpasses a certain threshold, the color of the sphere changes from white to purple and its size increases proportionally to the feedback parameter. If this lasts more than 2 seconds, the cube starts to rise until it reaches the top left corner. In this case, the feedback parameter was the relative amplitude in the UA band computed by equation (1). In this equation, *X*(*k*) denotes the frequency amplitude spectrum computed using the fast Fourier transform with a frequency resolution represented by Δ*f*, using a sliding window of 2 seconds and shifts every 0.125 seconds, as presented in [27]:

(1) $$\begin{array}{c} \text{UA Relative Amplitude}=\frac{\sum_{k=\text{IAF}/\Delta f}^{\text{HTF}/\Delta f}\;\;X\left(k\right)/\left(\text{HTF}-\text{IAF}\right)}{\sum_{k=4/\Delta f}^{30/\Delta f}\;\;X\left(k\right)/\left(30-4\right)}. \end{array}$$

Therefore, if the threshold is set to *x*, the subject will receive a positive feedback every time the UA amplitude is above *x* times the amplitude of the EEG from 4 to 30 Hz. All participants started with a threshold value of 1, which was found empirically to be a good starting point [13].

The feedback was continuous, and the threshold was adjusted according to individual performance, evaluated by the average percentage of time, for a set of blocks, during which the goal was reached. If the percentage of time during which the feedback parameter was above the threshold exceeded 60%, the threshold was increased by 0.1. If this percentage was lower than 20%, the threshold was decreased by 0.1. This was done in order to keep it challenging if the performance was considered good and, if the opposite happened, to allow the subject to find the most successful mental strategies without losing motivation along the process.

In the INTENSIVE group, between the sets of blocks, there was a larger break (of approximately 1-5 minutes) which was used to check the average time spent above the threshold and update the NF threshold if necessary. For the SPARSE group, threshold updating only occurred at the end of each session, to define which threshold to start with on the following session. Threshold updating differed between groups since we consider that a minimum number of blocks is needed to determine changes and guarantee that the achieved progress is stable.

*(2) Mental Strategies*. The participants should use a single mental strategy per block, so that the effects of that strategy could be isolated in order to rate its effectiveness. During the first session, they were encouraged to try different strategies in order to understand which ones produced better results and then repeat them afterwards. Although the participants were not encouraged to use any specific strategies, some examples were provided when they asked for them, based on Nan et al. [13].

Since they were allowed to choose the more suitable strategies for them, the preferentially applied strategies varied a lot. The most successful strategy of each session (which corresponded to the block with the highest UA amplitude) was collected for each participant. After gathering all the best strategies for every participant, 49 distinct strategies were found which were then grouped into six categories: “feedback,” related to feedback display and the screen; “imagination,” related to fantasizing about fictional episodes; “memories,” for recalling past experiences; “mental,” when performing tasks that involved mental effort; “motor,” when thinking about performing physical activities; and “relaxation,” when attempting to relax the body and mind (for example, with breathing exercises). For both groups, “relaxation” was the preferred category (INTENSIVE: 38.89% and SPARSE: 34.07%).

### 2.5. Questionnaires

In order to keep record of how mental state factors such as concentration, motivation, sleepiness, and stress affected training, a questionnaire to assess these factors was used. For this purpose, a rating scale was used to evaluate the frequency of the four mentioned states/sensations during training: 1—never, 2—rarely, 3—sometimes, 4—frequently, 5—always.

Furthermore, the 36-Item Short Form Survey (SF-36) questionnaire, which has been validated for the Portuguese population, was used in the first session before training to assess different domains of health state and quality of life [31, 32]. It was employed in order to ensure that the participants did not have abnormal health conditions.

### 2.6. Data Processing and Extraction

The first step was to remove artifacts that would mislead the analysis. EEG was bandpass filtered between 4 and 30 Hz, for low-frequency components of eye movement and high-frequency muscle artifact removal. This filtering, although it did not completely remove ECG artifacts, reduces significantly their main frequency components. Except for eye movements, the additional artifacts for all baseline measurements of each participant were removed manually, through visual inspection. This included the rejection of periods with muscle artifacts (higher amplitude and frequency, mostly frontally and temporally), sweat artifacts (undulating waves with low amplitude and longer duration than regular waves), electrode pop/movement artifacts (brief transients usually restricted to a single electrode) and, in general, artifacts of other segments with an amplitude much greater than the surrounding activity and remarkably different from brain-generated waveforms [33].

In the second step, for each training block of each subject, the relative amplitude of several bands of interest (shown in Table 1) was computed following the example of equation (1).

The analysis of the extracted data was performed using MATLAB software (version 2015b). The topographic distributions were generated using FieldTrip [34], an open-source MATLAB toolbox.

### 2.7. Evaluation of Training Performance

Participants have different baseline amplitude values (intervariability), which also vary when assessed in different days (intravariability). Therefore, for offline analysis, the relative amplitude values for each individual session were normalized. The normalization was enforced by dividing the relative amplitudes during NF by the corresponding relative amplitudes of the pre-training baseline with EO for that session. Therefore, for simplification, normalized relative amplitudes will be hereafter referred to as amplitudes only.

There is no standard approach to evaluate the training performance. Several authors relied on clusters of sessions or representative sessions, such as the first and the last, to extract conclusions about training effectiveness [23, 35–37]. On the other hand, others used measures that consider the progress along the whole training [13, 16]. In this case, in order to evaluate the evolution of the amplitude of frequency bands along time, both across sessions and within session, distinct indexes were used to capture different aspects. We derived two types of measures, one that explores the overall progress and another to take into account the small variations that may occur.

(i) Across sessions: the learning was assessed by *A*_diff_ and *A*_trend_, shown in equations (2) and (3), respectively, where *S* denotes the total number of sessions and *s*_*i*_ represents the *i*-th session
  (a) *A*_diff_: this refers to the amplitude change of the last two sessions relative to the first two sessions. Two sessions were used aimed at increasing robustness to outliers:
    (2) $$\begin{array}{c} A_{\text{diff}}=\frac{\left(s_{S-1}+s_{S}\right)-\left(s_{1}+s_{2}\right)}{\left(s_{1}+s_{2}\right)}. \end{array}$$
  (b) *A*_trend_: this corresponds to the slope of the linear trend line that illustrates the evolution of the amplitude across all sessions, so that the variations that may occur in between are taken into account. Taking *y* = *mx* + *b*, *A*_trend_ corresponds to *m*:
    (3) $$\begin{array}{c} A_{\text{trend}}=m, \end{array}$$
    with *y* corresponding to the relative amplitude of a certain frequency range, *x* representing the session number, and *b* standing for the *y*-intercept which relies on each participant's intrinsic characteristics.
(ii) Within session: the learning was assessed by *W*_diff_ and *W*_trend_, shown in equations (4) and (5), respectively, where for *S* sessions and *B* blocks, the *i*-th session is denoted by *s*_*i*_ and the *j*-th block of that session by *b*_*j*,*i*_
  (a) *W*_diff_: this measure is based on [27] and aimed at quantifying the changes in relation to the first block within session across all training sessions:
    (4) $$\begin{array}{c} W_{\text{diff}}=\frac{\sum_{i=1}^{S}\;\;\sum_{j=2}^{B}\;\;\left(b_{j,i}-b_{1,i}\right)}{S\times \left(B-1\right)}. \end{array}$$
  (b) *W*_trend_: it is the slope of the linear regression that describes the evolution of the amplitude of a certain frequency band along blocks, averaged across sessions. Considering *y* = *m*_*i*_*x* + *b*_*i*_ as the trendline for *s*_*i*_,
    (5) $$\begin{array}{c} W_{\text{trend}}=m_{\text{average}}=\frac{\sum_{i=1}^{S}\;\;m_{i}}{S}, \end{array}$$
    in which *y* corresponds to the amplitude, *m*_*i*_ is the slope, *x* is the block number, and *b*_*i*_ is the *y*-intercept, which will depend mostly on each subject's characteristics.

### 2.8. Statistical Analysis

The Shapiro-Wilk test [38] found that normality was not verified in all examined variables for NF training analysis. Therefore, we employed nonparametric tests in the following analyses.

In addition to the UA, we tested also neighbour bands (ITB, LA, and SMR) to assess whether the target band was trainable independently of a change in the other frequency bands [39]. To analyze the median amplitude of each frequency band within each group, the Wilcoxon signed-rank test was used [40]. For the UA amplitude evolution within and across sessions, right-tailed tests were employed due to a prior hypothesis of an increase.

In order to make comparisons of amplitudes between groups, the Wilcoxon rank-sum test was used to test if the medians considering all the participants of each group differed significantly [41]. A significance level of 5% was considered.

The topographical effects of training were also inspected in order to understand whether UA amplitude changes were restrained to the training location, Fz, or if there was a spread to other areas. In the case of the topographical analysis, since multiple comparisons were performed as there were 20 electrodes, the false discovery rate (FDR) was controlled with the Benjamini-Hochberg procedure [42]. For this purpose, the corrected *p* values were computed using the Multiple Testing Toolbox [43].

## 3. Results

### 3.1. NF Training

The analysis of NF training results focuses on the changes that occur both along the time course of the training experiment and within each session. On the one hand, the evolution of the UA amplitude was examined to assess trainability. On the other hand, with respect to independence, changes regarding other frequency bands are also shown in order to assess if the training effects were restricted to the target band [39].

#### 3.1.1. Across Sessions

**Figure 2:**
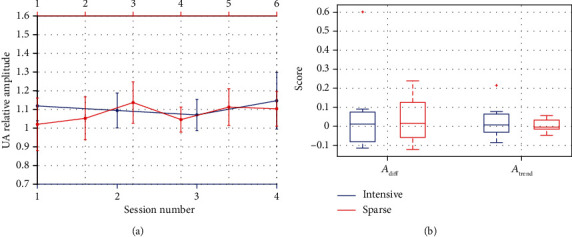
(a) Evolution across sessions for the INTENSIVE (blue) and SPARSE (red) groups during neurofeedback (NF) at Fz: median values of upper alpha (UA) band amplitude; error bars show median absolute deviation. (b) Boxplot with the distribution of *A*_diff_ (equation (2)) and *A*_trend_ (equation (3)) measures for the INTENSIVE (blue) and SPARSE (red) groups; + represents outliers.

*(1) Training Location: Fz*. The evolution of the UA amplitude across sessions for both groups is depicted in Figure 2(a), and the learning measures are represented in Figure 2(b). The results within group for all frequency bands are shown in Table 2.

For the UA amplitude, no significant effects across sessions were found for *A*_diff_ or *A*_trend_, either within groups or between them.

Regarding other frequency bands, within group, only for the SPARSE group, LA shows significant changes across sessions (*A*_diff_: *W* = 40; *p* = 0.039). The groups only differ significantly regarding SMR (*A*_trend_: *U* = 64; *p* = 0.040). For all the others, there were no significant differences for any of the learning indexes (*p* ≥ 0.050).

**Figure 3:**
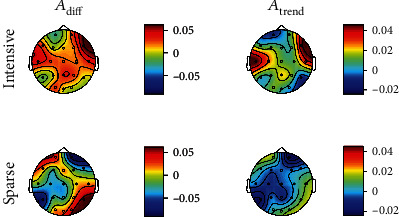
Topographic distribution of median *A*_diff_ (equation (2)) and *A*_trend_ (equation (3)) measures for the INTENSIVE and SPARSE groups across sessions for the upper alpha (UA) band.

*(2) Topographic Distribution*. Concerning the measures of performance across sessions, represented in Figure 3, there is no clear increase around Fz when compared to the other electrodes, for either the INTENSIVE or the SPARSE group. There are no significant differences between groups for any of the electrodes using corrected *p* values.

#### 3.1.2. Within Session

**Figure 4:**
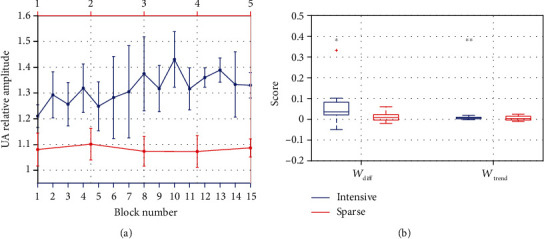
(a) Evolution within session for the INTENSIVE (blue) and SPARSE (red) groups during neurofeedback (NF) at Fz: mean of the upper alpha (UA) amplitude in all corresponding blocks considering the median of the participants; error bars show standard deviation. (b) Boxplot with distribution of *W*_diff_ (equation (4)) and *W*_trend_ (equation (5)) measures for the INTENSIVE (blue) and SPARSE (red) groups; + represents outliers; ∗ represents *p* values below 0.05 and ∗∗ represents *p* values below 0.01.

*(1) Training Location: Fz*. Despite the fact that the within-session performance might change along sessions, we analyzed them globally for all sessions. Therefore, Figure 4 represents the mean value for each block across sessions, considering the median for all participants. While there is no visible trend for the SPARSE group, the amplitude tends to increase along blocks for the INTENSIVE group, although it slightly decreases in the last two blocks. The evolution of the learning measures within session can be visualized in Figure 4. The results within group for all frequency bands are shown in Table 3.

For the UA band, there are no significant differences between groups within session, considering any of the learning indexes (*p* ≥ 0.050). However, both *W*_diff_ and *W*_trend_ for the UA in the INTENSIVE group are significantly larger than zero, while for the SPARSE group, neither *W*_diff_ nor *W*_trend_ showed significant difference from zero.

Regarding the other frequency bands, there are significant differences between groups for LA only (*W*_diff_: *U* = 65; *p* = 0.030), as for the rest of the frequency bands (*p* ≥ 0.050) for both of the learning indexes. For the INTENSIVE group, *W*_diff_ and *W*_trend_ are also significantly different from zero for LA. For the SPARSE group, neither *W*_diff_ nor *W*_trend_ showed significant differences from zero for any of the frequency bands.

Therefore, although only the INTENSIVE group reveals changes within session, these are not restricted to the target band and spread over the whole IAB.

**Figure 5:**
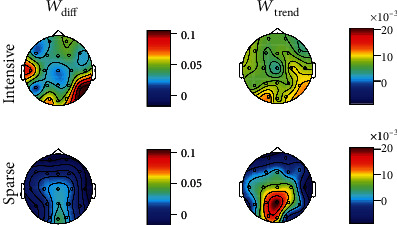
Topographic distribution of median of *W*_diff_ (equation (4)) and *W*_trend_ (equation (5)) measures for the INTENSIVE and SPARSE groups within session for the upper alpha (UA) band.

*(2) Topographic Distribution*. By comparing the topographic distributions in Figure 5, it is visible that, for both *W*_diff_ and *W*_trend_, the values are generally higher for the INTENSIVE group, specially in the occipital, temporal, and parietal areas. However, regarding *W*_trend_, the maximum value occurs for the SPARSE group, at Pz. Although not directly targeted by the training, an increase in the UA amplitude in posterior areas might be more easily promoted as the alpha waves occur predominantly in the occipital area. The measures do not significantly differ between groups for any of the electrodes, considering the corrected *p* values.

## 4. Discussion

Considering the whole training across sessions for all participants, none of the training intensity modalities revealed to be effective in producing an increase in the UA amplitude. Different results across sessions were obtained by Zoefel et al. [39] and Escolano et al. [16] for the enhancement of the UA band, yet they only considered the learners (79% and 60%, respectively). Both studies performed 5 sessions with 5 training blocks of 5 minutes each in five consecutive days. The training load in each session was equivalent to the one for the SPARSE group, but the training was done intensively. This suggests that shorter sessions on consecutive days may be more suitable for UA increase across sessions.

Regarding within session, the INTENSIVE group accomplished a good short-term training performance, whereas the SPARSE group did not. Yet, another study, also performing 5 trials with 5 minutes each, found a significant positive tendency for the UA (using a metric like *W*_trend_) [24]. Notwithstanding, only one session was performed, so it cannot be equitably compared with the present case (6 sessions). Nevertheless, although only the INTENSIVE group showed effects, the differences in the UA band between groups within session were not significant, not allowing concluding if one group effectively outperforms the other.

Furthermore, although there were changes in UA amplitude within session for the INTENSIVE group, there were no significant changes across sessions for any of the groups. As pointed out by Witte et al. [44], it might be the case that the training does not directly translate into a steady increase of the feedback parameter over time. Nonetheless, participants may learn how to master this skill and be able to do so, even if their brain activity is not altered over time.

Regarding the neighbour frequency bands, across sessions, the LA changed significantly for the SPARSE group only. Moreover, within session, while for the SPARSE group, there were no significant effects on other frequencies, for the INTENSIVE group, there were significant changes within session for LA. Hence, it is considered that UA was not trained independently from other frequency bands within session for this group. These results are not in line with Zoefel et al. [39] and Escolano et al. [16]. It is worth noticing again, however, that their analysis refers only to learners and was based on different computations. Nevertheless, it is possible that the changes in LA occur due to the demand for internalized attention of the NF training itself. An LA amplitude decrease is associated with alertness and vigilance. Contrarily, meditative states, which are associated with a switching off of these mechanisms, have been related to an increase in LA [45]. Therefore, the LA amplitude increase might have been caused by using strategies related to relaxation and mind emptiness, which were reported by the participants of both the INTENSIVE and SPARSE groups. Besides, although LA and UA may change individually, they are still closely interconnected as part of the IAB.

In this study, the focus was on NF learning and hence the baseline periods were used only to compute the IAB and not to derive conclusions on the effects of training intensity. Nevertheless, only a passive baseline was recorded, and perhaps future studies of NF training intensity should also include an active baseline [16, 24, 39, 46]. This should allow a better comparison between the baselines and NF periods, since the NF training involves active engagement from the participant. However, since the reported active tasks visually resemble the NF training, it cannot be discarded that, after the participant has experienced training, visualizing feedback elements may trigger changes in activity even if the training is not being applied.

Despite the review of the literature performed by Rogala et al. [9], to our knowledge, our study is the first that directly compares the effects of two distinct NF training intensity modalities through a practical experiment. Although the option of changing several variables (number of sessions, interval between sessions, and duration of each session) at the same time may prevent the isolation of a single effect, it allows a preliminary investigation of the impact of distinct intensity factors. The examination of only one intensity parameter would rely on the assumption of a fixed model dependent on a specific variable and would reduce the chances of finding any effects, as there are many possible sources of impact on training. Accordingly, as a preliminary study, we selected two protocols as distinct as possible regarding these three variables of training intensity, which would still be feasible in terms of total duration and availability of the participants. Thus, the actual choice of parameters represents a compromise between the contrast between groups and the feasibility of the experiment. As training intensity encompasses numerous parameters, more groups or distinct experiments would be needed to test the influence of each of them on training performance. Furthermore, single parameters of the training protocol should be systematically varied, to understand their specific contribution. Despite the within-session results for the INTENSIVE group, when further investigating intensive protocols, we should take care regarding overtraining, especially with vulnerable participants. For this purpose, a checklist of risk factors can be used, and the within-session progress may be examined to detect task fatigue [47].

In addition to the choice of particular training intensity parameters, another limitation is related to the relatively small sample size (with 9 participants in each group), lacking a randomized assignment to groups. To fully capture effects, a larger sample size would be desirable to achieve greater statistical power. Moreover, although the gender difference between the two groups was not significant, the gender was not balanced (77.8% females in the INTENSIVE group; 33.3% females in the SPARSE group). In future work, groups should be matched regarding age and gender, to avoid bias. Furthermore, the UA amplitude was extracted from the individual alpha band instead of a fixed range of values. This decision allows for a more personalized approach, which could facilitate NF training. Yet, it may also be a source of error and variability, as the measure relied on a single baseline measurement (during the first session only). Besides, there are different methods to estimate UA (e.g., Zoefel et al. [39] use IAF to IAF + 2 Hz, considering the IAF as the peak frequency of the alpha band), which often prevents a direct comparison with the literature that relies on individual values.

Some studies on clinical samples have observed lasting improvements several weeks [48] and even a year [49] after NF training. In the case of Rance et al. [48], the clinical improvement even grew over time after the NF intervention, though the electrophysiological variables are not reported. In the present case, we have not included a follow-up analysis. Therefore, ideally, future studies should also characterize the time course of NF responses over several months, to examine how training intensity may affect the preservation of long-lasting outcomes and better characterize the progress.

Moreover, it should be noted that this experiment was performed with healthy participants, and a specific band and training location were used. Choosing a clinical target or different protocols may cause significant changes regarding the suitability of a certain intensity modality. Additionally, a control group that would also allow assessing both physiological and behavioural changes in a reliable way is recommended to mitigate the impact of interindividual variations and allow for strongly supported conclusions.

## 5. Conclusions

In the present study, an experiment of NF training for the increase in the activity in the UA band was performed to compare the effects of two different training intensity modalities. Although further investigation is needed to establish if one modality outperforms the other, the results suggest that better outcomes might be obtained within session by adopting the intensive design. To the best of the authors' knowledge, there are no other studies focused only on training intensity, in which two groups with different training intensity modalities were compared or intensity parameters were exhaustively studied. Thus, it is considered that, although being exploratory, the present work introduces new findings in the field. In the future, we encourage the use of carefully detailed reports of training parameters, as this is a crucial step towards thoroughly studying NF training intensity.

## Figures and Tables

**Table 1:** Frequency bands.

Frequency bands	Frequency range (Hz)
Individual theta band (ITB)	4 to LTF
Individual lower alpha band (LA)	LTF to IAF
Individual upper alpha band (UA)	IAF to HTF
Sensorimotor rhythm (SMR)	12 to 15

**Table 2:** Within-group analysis across sessions.

Frequency bands	INTENSIVE						SPARSE					
	*W*_diff_			*W*_trend_			*W*_diff_			*W*_trend_		
	Mdn	*W*	*p*	Mdn	*W*	*p*	Mdn	*W*	*p*	Mdn	*W*	*p*
ITB	0.028	29	0.496	0	21	0.910	0.064	38	0.074	0.015	36	0.129
LA	-0.022	19	0.734	-0.004	18	0.652	0.094	40	**0.039**	0.022	36	0.129
UA	0.011	24	0.455	0.007	27	0.326	0.016	30	0.213	-0.004	27	0.326
SMR	0.009	34	0.203	0.010	36	0.129	-0.013	15	0.426	-0.007	11	0.203

Notes: median (Mdn), *W* statistic (*W*), and *p* values (*p*) resulting from the Wilcoxon signed-rank test (right-tailed for the UA band and two-tailed for the other frequency bands); the medians with absolute value below 0.001 are shown as zero.

**Table 3:** Within-group analysis within session.

Frequency bands	INTENSIVE						SPARSE					
	*W*_diff_			*W*_trend_			*W*_diff_			*W*_trend_		
	Mdn	*W*	*p*	Mdn	*W*	*p*	Mdn	*W*	*p*	*Mdn*	*W*	*p*
ITB	0.003	22	1.000	0	19	0.734	0	29	0.496	-0.003	16	0.496
LA	0.090	43	**0.012**	0.008	44	**0.008**	0.017	29	0.496	0.006	34	0.203
UA	0.036	40	**0.020**	0.006	43	**0.006**	0.008	33	0.125	0.003	34	0.102
SMR	-0.005	21	0.910	0	12	0.250	0.004	27	0.652	-0.001	24	0.910

Notes: median (Mdn), *W* statistic (*W*), and *p* values (*p*) resulting from the Wilcoxon signed-rank test (right-tailed for the UA band and two-tailed for the other frequency bands); the medians with absolute value below 0.001 are shown as zero.

## Data Availability

The data used for this study is available upon request to the corresponding author.

## Supplementary Materials

Supplementary Materials We present a summary of our methodology using the “Consensus on the reporting and experimental design of clinical and cognitive-behavioural neurofeedback studies” (CRED-nf checklist) [52]. This summary was generated using the standardized CRED-nf online tool version 1.0 (http://rtfin.org/CREDnf).
