# Supplementary material for: An Exploratory Study of Training Intensity in EEG Neurofeedback
Source: Neural Plast. 2021 Mar 11;2021:8881059. doi: 10.1155/2021/8881059 (PMC7979284; doi:10.1155/2021/8881059)
Supplement: Supplementary Materials — We present a summary of our methodology using the “Consensus on the reporting and experimental design of clinical and cognitive-behavioural neurofeedback studies” (CRED-nf checklist) [52]. This summary was generated using the standardized CRED-nf online tool version 1.0 (http://rtfin.org/CREDnf). [file 8881059.f1.pdf]

# CRED-nf checklist summary

*21 January, 2021*

**Manuscript title:** An exploratory study of training intensity in EEG neurofeedback
**Corresponding Author:** Wenya Nan
**Corresponding author email:** wynan1985@126.com

| Item No. | Checklist item | Manuscript Details |
|---|---|---|
| **Pre-experiment** | | |
| 1a | Pre-register experimental protocol and planned analyses | *This experiment was not preregistered* |
| 1b | Justify sample size | *The manuscript does not describe the sampling plan or justify the sample size used* |
| **Control groups** | | |
| 2a | Employ control group(s) or control condition(s) | *This experiment did not include a control group or control condition* |
| 2b | When leveraging experimental designs where a double-blind is possible, use a double-blind | *NA: A double-blind was not appropriate for this experiment* |
| 2c | Blind those who rate the outcomes | *Those who rated the outcome were not blind to group assignment* |
| | Blind those who analyse the data | *Those who analysed the data were not blind to group assignment* |
| 2d | Examine to what extent participants and experimenters remain blinded | *No measures were taken to examine whether participants and experimenters remained blind* |
| 2e | In clinical efficacy studies, employ a standard-of-care intervention group as a benchmark for improvement | *NA: This is not a clinical efficacy study* |
| 3a | Collect data on psychosocial factors | The 36-Item Short Form Survey (SF-36) questionnaire was performed to assess general health state in the first session (before training). In every session, participants were also asked to fill a questionnaire in which they rated several parameters referring to their mental state during the session. (...) In order to keep record of how mental state factors such as concentration, motivation, sleepiness and stress affected training, a questionnaire to assess these factors was performed. For this purpose, a rating scale was used to evaluate the frequency of the four mentioned states/sensations during training: 1 - never, 2 - rarely, 3 - sometimes, 4 - frequently, 5 - always. |

| | | |
|---|---|---|
| 3b | Report whether participants were provided with a strategy | Although the participants were not encouraged to use any specific strategies, some examples were provided when they asked for them, based on Nan et al. [14]. |
| 3c | Report the strategies participants used | After gathering all the best strategies for every participant, 49 distinct strategies were found which were then grouped into six categories: ”feedback”, related with feedback display and the screen; ”imagination”, related with fantasizing about fictional episodes; ”memories”, for recalling past experiences; ”mental”, when performing tasks that involved mental effort; ”motor”, when thinking about performing physical activities; ”relaxation”, attempting to relax the body and mind (for example, with breathing exercises). |
| 3d | Report methods used for online-data processing and artifact correction | [online] The signals were submitted to notch filtering (50 Hz) and low pass filtering (30 Hz) (...) [offline] The first step was to remove some recorded periods which contained artifacts that would mislead the analysis. EEG was bandpass filtered between 4 to 30Hz, for low frequency components of eye movement and high frequency muscle artifacts removal. This filtering, although not completely removes ECG artifacts, reduces significantly their main frequency components. Except for eye movements, the additional artifacts for all baseline measurements of each participant were removed manually, through visual inspection. This included the rejection of periods with: muscle artifacts (higher amplitude and frequency, mostly frontally and temporally), sweat artifacts (undulating waves with low amplitude and longer duration than regular waves), electrode pop/movement artifacts (brief transients usually restricted to a single electrode) and, in general, of other segments with an amplitude much greater than the surrounding activity and remarkably different from brain-generated waveforms [34]. |
| 3e | Report condition and group effects for artifacts | *Condition and group effects for artifacts were not measured, or not reported in the manuscript* |

| | | |
|---|---|---|
| 4a | Report how the online-feature extraction was defined | The first baseline measurements of the first session were used to define the individual alpha band (IAB) of each individual, based on the difference between EO and EC spectra [28, 29]. The signals were submitted to notch filtering (50 Hz) and low pass filtering (30 Hz) and the power spectrum density was estimated using the Welch's method [30], with an overlap of 10 % and a segment length of 5 seconds. The crossings between EO and EC spectra provided the frequency boundaries: Lower Transition Frequency (LTF) and Higher Transition Frequency (HTF) [14]. If the crossings were not clearly visible from the spectra at Fz, we investigated occipital electrodes, where the alpha activity is usually more pronounced [21]. The individual UA band was defined as the frequency range between the individual peak alpha frequency and the HTF. The individual peak alpha frequency was defined as the frequency with largest power in the range 7.5-12.5 Hz in the EC power spectra and was considered equivalent to the Individual Alpha Frequency (IAF). The frequency range obtained for each participant was used for the online feedback and the subsequent offline analysis. (...) In this case, the feedback parameter was the relative amplitude in the UA band computed by Equation (1). Therefore, if the threshold is set to x, the subject will rec |
| | | eive e a positive feedback every time the UA amplitude is above x times the amplitude of the EEG from 4 to 30 Hz. All participants started with a threshold value of 1, which was found empirically to be a good starting point [14]. |
| 4b | Report and justify the reinforcement schedule | The feedback was continuous and the threshold was adjusted according to individual performance, evaluated by the average percentage of time, for a set of blocks, during which the goal was reached. If the percentage of time during which the feedback parameter was above threshold exceeded 60%, the threshold was increased by 0.1. If this percentage was lower than 20%, the threshold was decreased by 0.1. This was done in order to keep it challenging if the performance was considered good and, if the opposite happened, to allow the subject to find the most successful mental strategies without losing motivation along the process. In the INTENSIVE group, between the sets of blocks there was a larger break (of approximately 1-5 minutes) which was used to check the average time spent above threshold and update the NF threshold if necessary. For the SPARSE group, threshold updating only occurred at the end of each session, to define which threshold to start with on the following session. Threshold updating differed between groups since we consider that a minimum number of blocks is needed to determine changes and guarantee that the achieved progress is stable. |

| | | |
|---|---|---|
| 4c | Report the feedback modality and content | The EEG training platform integrated in Somnium software was adopted to perform NF training, using a visual feedback modality with the display described in more detail in Rodrigues et al. [16]. This display uses two three-dimensional objects against a grey background: a white/purple sphere, in the center, and a blue cube, in the lower left corner. These shapes suffer changes during the training, reacting to the participant's EEG in real time, according to previously defined settings. If the feedback parameter surpasses a certain threshold, the color of the sphere changes from white to purple and its size increases proportionally to the feedback parameter. If this lasts more than 2 seconds, the cube starts to rise until it reaches the top left corner. |
| 4d | Collect and report all brain activity variable(s) and/or contrasts used for feedback, as displayed to experimental participants | Our focus is on studying the learning of EEG regulation (i.e., NF learning), which in this case is learning to increase the amplitude of the UA amplitude, and how it is affected by training intensity. The NF learning was assessed by examining the changes in amplitude of UA across sessions and within session. (...) In this case, the feedback parameter was the relative amplitude in the UA band computed by Equation (1). |
| 4e | Report the hardware and software used | The acquisitions were carried out using Somnium software [18], in a room provided by the Evolutionary Systems and Biomedical Engineering Lab (LaSEEB), a research lab of Institute for Systems and Robotics (ISR), at Instituto Superior Técnico (IST), University of Lisbon. Electrodes were placed according to the International 10-20 System, using the left and right mastoids as references for common mode rejection and the middle of the forehead as ground. Relevant signal was recorded, with a sampling frequency of 250 Hz, from 20 electrodes: Fz, Fp1, F7, F3, T3, C3, T5, P3, O1, Cz, Pz, Oz, Fp2, F8, F4, T4, C4, T6, P4 and O2, and amplified by the EEG amplifier Vertex 823 (produced by Meditron Electromedicina Ltda, São Paulo, Brazil), with an analog band-pass filter between 0.1 and 70 Hz. The impedance of each electrode was kept below 10 kOhm. (...) The analysis of the extracted data was performed using MATLAB software (version 2015b). The topographic distributions were generated using Fieldtrip [35], an open-source MATLAB toolbox. |
| 5a | Report neurofeedback regulation success based on the feedback signal | The evolution of the UA amplitude across sessions for both groups is depicted on Figure 2 (left) and the learning measures are represented on Figure 2 (right). The results within group for all frequency bands are shown in Table 2. For the UA amplitude, no significant effects across sessions were found for Adiff or Atrend, either within groups or between them. (...) Figure 4 represents the mean value for each block across sessions, considering the median for all participants. (...) The evolution of the learning measures within session can be visualized in Figure 4. The results within group for all frequency bands are shown in Table 3. For the UA band, there are no significant differences between groups within session, considering any of the learning indexes ($p >= 0.050$). However, both Wdiff and Wtrend for the UA in the INTENSIVE group are significantly larger than zero. While for the SPARSE group neither Wdiff nor Wtrend showed significant difference from zero. |

| | | |
|---|---|---|
| 5b | Plot within-session and between-session regulation blocks of feedback variable(s), as well as pre-to-post resting baselines or contrasts | between-session: Figure 2; within-session: Figure 4; |
| 5c | Statistically compare the experimental condition/group to the control condition(s)/group(s) (not only each group to baseline measures) | *The manuscript does not statistically compare the experimental condition/group to the control condition(s)/group(s)* |
| **Outcome measures - behaviour** | | |
| 6a | Include measures of clinical or behavioural significance, defined a priori, and describe whether they were reached | *The manuscript does not include measures of clinical or behavioural significance* |
| 6b | Run correlational analyses between regulation success and behavioural outcomes | *This manuscript does not compare regulation success and behavioural outcomes* |
| **Data storage** | | |
| 7a | Upload all materials, analysis scripts, code, and raw data used for analyses, as well as final values, to an open access data repository, when feasible | *No additional documents related to the materials, analysis scripts, code, raw data, or final values are available for this manuscript* |
